# Prevalence of depression, its correlates among students, and its effect on health-related quality of life in a Turkish university

**DOI:** 10.1080/03009730903174339

**Published:** 2009-09-07

**Authors:** Gul Arslan, Unal Ayranci, Alaettin Unsal, Didem Arslantas

**Affiliations:** ^1^Eskisehir Osmangazi University, Health Services Vocational High School26480, Meselik-EskisehirTurkey; ^2^Osmangazi University, Medico-Social Center26480, Meselik-EskisehirTurkey; ^3^Eskisehir Osmangazi University, Medical Faculty, Public Health Department26480, Meselik-EskisehirTurkey

**Keywords:** Depression, quality of life, Turkey, university students

## Abstract

**Objective:**

The aims were to investigate the prevalence of depression among university students, and to determine some of the risk factors connected to depression, and also to evaluate its effect on health-related quality of life (HRQoL).

**Methods:**

This cross-sectional survey was conducted between 1 December 2007 and 31 January 2008 at Osmangazi University, Eskisehir, in western Turkey. The study group consisted of 822 students. The questionnaire included the students’ socio-demographic characteristics, the Beck Depression Inventory, and the Medical Outcomes Study Short Form-36 (SF-36). The data were analyzed by using chi-square, Student's *t* test, percent (%) ratios, and backward logistic regression analysis with a significant value of *P<*0.05.

**Results:**

Of the students, 377 (45.9%) were males and 445 (54.1%) females. Overall, the prevalence of depression was 21.8% (*n*=179/822). Family history of depression, acne on face, any physical defect on body, smoking, alcohol consumption, and future-related occupational preoccupation were all deemed important risk factors for depression (*P <*0.05, for each one). It was found that, in those with depression, all the mean domain scores of SF-36 scale were lower than those without depression (*P <*0.05, for each one).

**Conclusions:**

The prevalence of depression among the university students in this region of Turkey was wide-spread, affecting negatively the HRQoL of the students. For prevention and control of depression, depression information and knowledge need to be addressed by health education programs.

## Introduction

Depression is a common mental disorder that presents with depressed mood, loss of interest or pleasure, feelings of guilt or low self-worth, disturbed sleep or appetite, low energy, poor concentration, and tendency to suicide, which can be seen in anybody regardless of age, gender, race, or socio-economic status (1).

The period of youth is a time of contradictions when a person goes through many changes and experiences such as emotional, behavioral, sexual, economic, academic, and social, and as well as efforts of discovering one's identity with psycho-social and sexual maturation. During this period, the mental health of university youth constitutes one of the important components of social health (2).

Psychological problems such as depression have significant implications for students’ lives, academic performance, and behavior. Students who reported experiencing at least one period of depression-like symptoms were significantly more likely to experience academic problems than were those without symptoms, in terms of receiving a lower grade (3).

Students reporting depression were significantly more likely to report less satisfaction with health-related quality of life (HRQoL) than students not reporting depression symptoms. Poor class-room performance is proportional to the daily frequency of students’ depression symptoms. Students who had depression symptoms have a significantly greater loss of healthy days compared to students who did not (4).

The prevalence of depressive symptoms varies across different populations. Specially, depressive symptoms are frequent among university students all over the world and their prevalence appears to be increasing (5). The ‘Turkey Mental Health Profile Project’ reported that depression was among the most frequently seen mental illnesses (6), and the prevalence of depressive symptoms in Turkish university students varied between 10% and 40% (7,8). Another study in the mid 1990s specified the prevalence rate at 34.5% (9), indicating an increase in depression among young adults in Turkey in the second half of the 1990s. We can speculate that changing environmental factors in the second half of the last decade negatively affected the psychological well-being of young people in Turkey.

This article presents data from a study of the students of a state university in a province of western Turkey, Eskisehir. The present cross-sectional study sought to address several areas of the subject: the depression status of Turkish university students, the socio-demographic factors affecting prevalence, and the effect on HRQoL.

## Methods

### Development of the questionnaires and instrument

The questionnaire consisted of three parts: in the first part of the questionnaire, the students were asked to state their demographic and medical characteristics. The second part of the questionnaire evaluated the status and the prevalence of depression. Depression was measured with a Turkish version of the Beck Depression Inventory (BDI) (10), which consists of 27 items. The BDI was developed by Beck et al. in 1961 (10) and later modified by Hisli in 1999 to suit the Turkish culture and norms (11). The answer for each item was evaluated as 0, 1, and 2 points. The lowest number of points was accepted as ‘0’ and the highest ‘54’, with a cut-off point of 19.

The third part of the questionnaire included the Medical Outcomes Study Short Form-36 (SF-36) instrument, which was used to determine the HRQoL of the students. The original questionnaire was developed by Ware and Sherbourne in 1992 (12), and its reliability and validity study for the Turkish version was tested by Kocyigit et al. (13), who showed good reliability and validity of this instrument in the Turkish validation study. The subjects gave appropriate answers for the questions in the SF-36 scale for their depression status during the last 4 weeks. Scores ranged from 0 to 100 for each domain separately.

### Participants

Eskisehir is a semi-rural province situated in the western part of Turkey, with a population of about 705,000. At Osmangazi University located in the province where the study was conducted, 8175 students were studying, and there were 6 schools (medicine, engineering and architecture, science and literature, economics, education, and the college of health services). Participants were determined with a two-step sampling method. In the first step, three schools were randomly selected, namely the schools of engineering and architecture, science and literature, and economics, the total student number of which was 6371. In the second step, at least one class from the first, second, third, and fourth years in each department were randomly selected to participate in this study. The study was restricted to the 822 (80.8%) accepting participants in the study, out of a total of 1018 students.

### Procedures

Following the completion of the questionnaires and inventories, each student's body mass index (BMI) was measured with domestic scales and height with a meter rule, and BMI was calculated using the formula, BMI=(weight (kg))/(height (m))^2^. Those whose BMIs were 25 kg/m^2^ and over were evaluated as overweight or obese (14). Students were also examined for the existence of acne through physical inspection.

The permission for the study was obtained by making a petition prior to collecting data by contacting and receiving approval from the Director of the Institution of Eskisehir Osmangazi University. Participants were assured of the confidentiality of their responses and provided informed verbal consent.

### Statistics

The statistical analysis was carried out using the Student's *t* test for continuous variables and the chi-square test for categorical variables. Significantly related variables were assessed in a model with logistic regression (stepwise backward Wald regression). Goodness-of-fit was calculated with the Hosmer-Lemeshow *c* statistic. Results are given as numbers and percentages (%) with 95% confidence interval (CI). A value of *P <*0.05 was considered statistically significant.

## Results

Of a total 822 participants, 377 (45.9%) were male and 445 (54.1%) female students. The average age of the participants was 20.82±1.83 years (range 17–30 years). There was no average age difference between male and female students (19.17±1.12, 19.85±1.36, respectively) (*P >*0.05). More than 40% of the students (40.6%) were from the school of engineering and architecture. Most students (67.2%) were in their freshman (44.0%) or sophomore (23.2%) years. A total of 25 (3.0%) and 52 (6.3%) of the students reported that their mothers and fathers, respectively, had died. The number of the students whose parents were divorced or were living separately was 54 (6.6%). The proportion of students whose mothers had an education level of primary school and lower was 49.5% (*n*=403), with the figure of 29.9% (*n*=246) reported for students’ fathers. Most students’ mothers were housewives (76.5%, *n*=629), with the proportion of 3.4% (*n*=28) reporting their fathers’ unemployment. Altogether 89.9% of the students (*n*=739) had a family structure of nucleus type, whereas 10.1% (*n*=83) had a family structure of patriarchal type. The mean number of the respondents’ siblings (brothers or sisters) was 2.71±1.6, ranging between 0 and 9. There were eight students with no siblings.

Most students (34.4%) reported staying with his/her house friends. Most students (36.6%) declared studying at their preferred departments, followed by failing to qualify for other departments requiring higher scores (33.2%). The prevalence of depression was significantly lower in those who were studying at their preferred departments (16.6%) when compared to the other reasons of choice (*P <*0.05).

It was reported that 57.4% of the students had a future-related occupational preoccupation. Those students had significantly higher prevalence of depression when compared to those did not have such a preoccupation (25.2% and 17.1%, respectively) (*P <*0.05).

The average score that the students obtained from the BDI was 11.21±8.56, ranging from 0 to 51. The students’ prevalence of depression was found to be 21.8% (*n*=179), with no statistically significant difference between male and female students (*P >*0.05). More detailed socio-demographic characteristics of those with and without depression are shown in Table I.

**Table I.** Some socio-demographic characteristics of students by status of depression.

	Depression			Statistical analysis
Socio-demographic characteristics	No *n* (%) 643 (78.2)	Yes *n* (%) 179 (21.8)	Total *n* (%) 822 (100.0)	Chi-square; *P*
Type of school				
Science and literature	249 (81.4)	57 (18.6)	306 (37.3)	3.622; 0.163
Economics	135 (74.2)	47 (25.8)	182 (22.1)	
Engineering and architecture	259 (77.5)	75 (22.5)	334 (40.6)	
Year in school				
Freshman	288 (79.6)	74 (20.4)	362 (44.0)	1.130; 0.770
Sophomore	149 (78.0)	42 (22.0)	191 (23.2)	
Junior	103 (78.0)	29 (22.0)	132 (16.1)	
Senior	103 (75.2)	34 (24.8)	137 (16.7)	
Age group (year)				
≤ 19	161 (76.3)	50 (23.7)	211 (25.7)	3.393; 0.414
20	143 (80.3)	35 (19.7)	178 (21.7)	
21	134 (79.3)	35 (20.7)	169 (20.65)	
22	91 (82.7)	19 (17.3)	110 (13.4)	
≥ 23	114 (74.0)	40 (26.0)	154 (18.7)	
Sex				
Male	288 (76.4)	89 (23.6)	377 (45.9)	1.371; 0.242
Female	355 (79.8)	90 (20.2)	445 (54.1)	
Living place				
With his/her family	180 (81.8)	40 (18.2)	220 (26.8)	4.219; 0.377
With his/her house friends	212 (74.9)	71 (25.1)	283 (34.4)	
Alone	31 (75.6)	10 (24.4)	41 (5.0)	
In dormitory	190 (78.5)	52 (21.5)	242 (29.4)	
Other (with any family, pension, hotel)	30 (83.3)	6 (16.7)	36 (4.4)	
Reason for study choice				
Own preference	251 (83.4)	50 (16.6)	301 (36.6)	12.424; 0.014
Failing to qualify for other department	206 (75.5)	67 (24.5)	273 (33.2)	
Family preference	27 (67.5)	13 (32.5)	40 (4.9)	
Having better job facilities	124 (79.5)	32 (20.5)	156 (19.0)	
Other (parent profession, advice by someone outside family, behave flamboyantly, marry a beautiful girl or a handsome one)	35 (67.3)	17 (32.7)	52 (6.3)	
Future-related occupational preoccupation				
Yes	353 (74.8)	119 (25.2)	472 (57.4)	7.682; 0.006
No	290 (82.9)	60 (17.1)	350 (42.6)	

In this study, the prevalence of smoking was 47.6% (*n*=391), with the prevalence of 36.7% (*n*=302) reported for alcohol consumers. The proportion of the students with a chronic disease diagnosed by a physician was 15.6% (*n*=128). There were some defects in about 10.0% of the students (*n*=80; 9.7%). Their distributions were as follows: visual impairments (*n*=62; 77.5%), hearing problems (*n*=3; 11.25%), orthopedic defects (*n*=9; 11.25%), and other defects (*n*=6; 7.5%).

The number of those with acne on physical examination was 330 (40.1%). The average BMI of the participants was 21.28±2.91 kg/m^2^ (range 14–33). There was no relationship between those who were overweight/obese and those who were not (21.89±3.01 and 19.97±2.86, respectively) in connection with depression (*P >*0.05). The prevalence of overweight and obese students was 9.7% (*n*=80). The number of those with family history of depression was 130 (15.8%). More detailed individual characteristics of students by status of depression are given in Table II.

**Table II.** Individual characteristics of students by status of depression.

	Depression			Statistical analysis
Individual characteristics	Yes *n* (%) 643 (78.2)	No *n* (%) 179 (21.8)	Total *n* (%) 822 (100.0)	Chi-square; *P*
Smoking habit				
Yes	280 (71.6)	111 (28.4)	391 (47.6)	19.142; 0.000
No	363 (84.2)	68 (15.8)	431 (52.4)	
Alcohol consumption				
Yes	217 (71.9)	85 (28.1)	302 (36.7)	11.370; 0.001
No	426 (81.9)	94 (18.1)	520 (63.3)	
Any chronic disease				
Yes	91 (71.1)	37 (28.9)	128 (15.6)	4.525; 0.033
No	552 (79.5)	142 (20.5)	694 (84.4)	
Any physical defect				
Yes	52 (65.0)	28 (35.0)	80 (9.7)	9.098; 0.003
No	591 (79.6)	151 (20.4)	742 (90.3)	
Acne vulgaris on face				
Yes	243 (73.6)	87 (26.4)	330 (40.1)	6.812; 0.009
No	400 (81.3)	92 (18.7)	492 (59.9)	
Overweight/obese				
Yes	64 (80.0)	16 (20.0)	80 (9.7)	0.164; 0.685
No	579 (78.0)	163 (22.0)	742 (90.3)	
Family history of depression				
Yes	89 (68.5)	41 (31.5)	130 (15.8)	8.640; 0.003
No	554 (80.1)	138 (19.9)	692 (84.2)	

A total of 739 (89.9%) students, whose prevalence of depression was 21.4%, were from nucleus family type, whereas only 83 students were from patriarchal family type (*P >*0.05). Although those with a patriarchal family type, one or more siblings, whose mothers were alive, whose fathers were not alive, whose parents were separated, having a parent with educational levels of secondary school or higher, whose mothers did not have a job, or whose fathers did not have a job showed higher proportions of depression compared to the other groups, they revealed no significant difference (*P >*0.05, each one). The only variable which affected depression was whether the students’ mothers had a job: in those whose mothers had a job the prevalence of depression was 27.5%, whereas in those whose mothers did not have a job this rate was 20.0% (*P <*0.05). The other family characteristics of the students with and without depression are presented in Table III.

**Table III.** Family characteristics of the students with and without depression.

	Depression			Statistical analysis
Family characteristics	No *n* (%)	Yes *n* (%)	Total *n* (%)	Chi-square; *P*
Family structure				
Nucleus	581 (78.6)	158 (21.4)	739 (89.9)	0.673; 0.412
Patriarchal	62 (74.7)	21 (25.3)	83 (10.1)	
Number of siblings				
0	8 (100.0)	0 (0.0)	8 (1.0)	3.356; 0.340
1–2	332 (79.4)	86 (20.6)	418 (50.8)	
3–4	258 (76.8)	78 (23.2)	336 (40.9)	
≥ 5	45 (75.0)	15 (25.0)	60 (7.3)	
Mother is alive				
Yes	622 (78.0)	175 (22.0)	797 (97.0)	0.505; 0.477
No	21 (84.0)	4 (16.0)	25 (3.0)	
Father is alive				
Yes	605 (78.6)	165 (21.4)	770 (93.7)	0.863; 0.353
No	38 (73.1)	14 (26.9)	52 (6.3)	
Parents are living separately				
Yes	42 (77.8)	12 (22.2)	54 (6.6)	0.007; 0.935
No	601 (78.3)	167 (21.7)	768 (93.4)	
Mother's educational level				
Primary school or below	325 (80.6)	78 (19.4)	403 (49.0)	2.721; 0.099
Secondary school or over	318 (75.9)	101 (24.1)	419 (51.0)	
Father's educational level				
Primary school or below	200 (81.3)	46 (18.7)	246 (29.9)	1.951; 0.162
Secondary school or over	443 (76.9)	133 (23.1)	576 (70.1)	
Mother has got a job				
Yes	140 (72.5)	53 (27.5)	193 (23.5)	4.785; 0.029
No	503 (80.0)	126 (20.0)	629 (76.5)	
Father has got a job				
Yes	622 (78.3)	172 (21.7)	794 (96.6)	0.177; 0.674
No	21 (75.0)	7 (25.0)	28 (3.4)	

According to the bivariate analysis results, significant differences were revealed between the existence of depression and the students’ reasons for preference of the schools, their future-related occupational preoccupation, smoking habit, alcohol consumption, presence of any chronic disease diagnosed by a physician, any physical defect, acne on face, family history of depression, and whether their mothers had a job. Backward stepwise logistic regression analysis formed with the above variables, which showed significantly important findings, is given in Table IV. According to this analysis, family history of depression (odds ratio (OR) 1.649), acne on face (OR 1.628), any physical defect (OR 2.043), smoking habit (OR 1.898), and a future-related occupational preoccupation (OR 1.690) were significantly important risk factors for depression (*P <*0.05, each one).

**Table IV.** Logistic regression analysis results formed by some variables considered as related to depression.

	Parameter estimates (β)	SE	Wald	df	P	OR	95% CI
Family history of depression (reference: no)							
Yes	0.500	0.219	5.193	1	0.023	1.649	1.072–2.535
Acne vulgaris on face (reference: no)							
Yes	0.488	0.178	7.497	1	0.006	1.628	1.149–2.309
Any physical defect (reference: no)							
Yes	0.715	0.261	7.486	1	0.006	2.043	1.225–3.409
Alcohol consumption (reference: no)							
Yes	0.361	0.194	3.459	1	0.063	1.435	0.981–2.100
Smoking habit (reference: no)							
Yes	0.641	0.194	10.867	1	0.001	1.898	1.297–2.779
Future-related occupational preoccupation (reference: no)							
Yes	0.525	0.183	8.182	1	0.004	1.690	1.180–2.421
Constant	−2.467	0.218	128.467	1	0.000	0.085	

Hosmer-Lemeshow test: chi-square = 8.646, df = 8; *P =*0.373. CI = confidence interval; df = degree of freedom; OR = odds ratio; SE = standard error.

It was determined that the means of all the domain scores that the students obtained from the SF-36 scale were significantly lower in those with depression compared to those without depression (*P <*0.001, for each domain). Table V shows the mean scores of SF-36 domains of those with depression and of those without.

**Table V.** Mean scores of SF-36 domains of those with and without depression.

	SF-36 score Depression		Statistical analysis
Domains	Yes (*n*=179) (mean±SD)	No (*n*=643) (mean±SD)	*t* test; *P*-value
Physical functioning	72.29±24.29	85.51±18.01	7.959; 0.000
Role—physical	58.66±39.24	76.79±33.70	6.133; 0.000
Bodily pain	62.30±24.68	71.83±20.57	5.239; 0.000
General health perception	50.53±18.93	62.47±17.22	8.028; 0.000
Vitality	41.68±19.62	58.59±16.46	11.640; 0.000
Social functioning	57.05±24.72	71.54±20.56	7.962; 0.000
Role—emotional	38.18±41.52	59.10±41.79	5.933; 0.000
Mental health	42.19±18.03	61.67±15.69	14.205; 0.000

SF-36 = Medical Outcomes Study Short Form-36.

## Discussion

Our results indicated that over one in five students (21.8%) had depression. Some studies in our country have reported that the prevalence of depression for university students was found to be between 10.0% and 40.0% (15,16), compatible with our study result. In parallel, in some studies on university students conducted in several countries, the prevalence of depression was observed to be between 8.3% and 45.0% (5,17). The aforementioned studies show that the rate of depression in university students ranges from 8.0% to 40.0%. While our result was higher than some study results, it was lower than others. One explanation for these differences in reported depression rates could be the inconsistency in how questions were asked regarding time-frame. A further possibility could be relevant to individuals’ different socio-demographic characteristics.

In those who studied at their preferred schools, the prevalence of depression was significantly lower than in those who studied at their schools for other reasons (*P <*0.05). This status is an expected result since they achieved their aims.

In various studies, an important relationship was found between occupational preoccupation with the future and job choice, and the occurrence of depression (15). These results are in line with our study results in both bivariate analysis (*P <*0.05) and multivariate analysis (OR 1.690; 95% CI 1.180–2.421) showing that there are important connections between occupational preoccupation and occurrence of depression (*P <*0.05).

There is some evidence that nicotine has antidepressant properties, which may explain the relationship that depressed persons may self-medicate by smoking (18). In our study, in the smoker students, the prevalence of depression was found to be significantly higher than in those not smoking (*P <*0.05). Many researchers support our study findings (5). One explanation for this could be that since the level of stress and anxiety in university students is rather high due to a stressful environment, a low allowance, and an intensive study tempo, they may have smoked more in line with the study showing that smokers with anxiety disorders reported greater anxiety sensitivity, anxiety symptoms, agoraphobic avoidance, depressed mood, negative affect, stress, and life interference, when compared to non-smokers (19).

Some evidence linking depression and alcohol abuse/dependence suggests that alcohol use and depression may be linked because persons who suffer from one disorder are prone to the other (20). These possibilities are compatible with our study result showing that the prevalence of depression was higher in the students consuming alcohol than in those who did not (*P <*0.05). Similarly, logistic model results showed a significant relationship between alcohol consumption and depression (OR 1.435; 95% CI 0.981–2.100), consistent with some studies (5,21). Because heavy alcohol use and daily smoking are each associated with depression, people who do both may be at an increased risk for depression. This is a public health issue because people who drink alcohol often also smoke and vice versa.

In the current study, in the students with any chronic disease, the occurrence of depression was more than in those without (*P <*0.05). Our finding is compatible with findings of a study (16). In that study, it was shown that disease inflicted a significant burden on the daily physical activities of the patients, as well as on their schooling and job opportunities. In addition to these problems, these students were also insensitively teased by peers because of their typical features, and those diseases had their own consequences on the students’ social behavior in that they were more introverted (16,22).

In the present survey, in both bivariate and multivariate analyses, any physical defect that the students had was an important risk factor for the occurrence of depression (*P <*0.05 and OR 2.043; 95% CI 1.225–3.409, respectively). This may be explained by the finding that individuals having an obsessive preoccupation with an imagined appearance defect feel potential social rejection (23).

In this study, in both bivariate and multivariate analyses, the students with acne on their face had a significantly higher risk for depression when compared to those who did not (*P <*0.05 and OR 1.628; 95% 1.149–2.309, respectively). By way of an explanation for this, it has been suggested that acne affects psycho-social health negatively due to the psychological issues attached to it, which include pain and discomfort, shame, body image, social assertiveness, obsessive-compulsiveness, embarrassment, and social inhibition. Furthermore, acne is also associated with a greater psychological burden than a variety of other disparate chronic disorders (24).

In this study, the prevalence of depression in the students who had a family history of depression was significantly higher than in those without (*P <*0.05). Similarly, the model showed the same significant connection (OR 1.649; 95% 1.072–2.535). This may be explained with the genetic epidemiology data suggesting that younger age of onset is associated with family history of depression (25).

This study found that in the students whose mothers had a job the prevalence of depression was higher than in those whose mothers did not (*P <*0.05), whereas there was no connection between those whose fathers had a job and whose fathers did not have a job in terms of the prevalence of depression (*P >*0.05). Whether parents have a job or not is a factor which directly affects the income level of family. Income level has a direct effect, both positive and negative, on social status and mental health of parents and children. Mothers who were employed, married, or both, reported better well-being than mothers who were both unemployed and unmarried, especially when their offspring had relatively higher adaptive functioning. This relationship between role occupancy and well-being was fully mediated by socio-economic status (SES). Sjöberg et al. reported that there was an important positive connection between depression and parents having job (26).

In our study, it was found that the HRQoL of students with depression was worse than for those without. According to the SF-36 scale, their HRQoL was seen to have been affected negatively in all the domains (*P <*0.001, for each domain). Similar results have been reported by many studies pointing out that HRQoL of individuals with depression was affected in a negative way (27). In a study by Gostautas et al. it was reported that the most frequently affected domain for those with depression was of physical functioning according to the SF-36 scale (28). Hayman et al. indicated that in those with depression the domains of physical functioning and mental health were affected in a negative way when compared to the other domains (23).

One of the limitations of this study was that it was cross-sectional, thus precluding inferences of casualty among variables. The second limitation is the self-reported nature of this study. Finally, the sample of the current study comprised a group of students in just one province of Turkey, which may limit generalization of the results through the other students. Thus, in order to definitively answer this question university students, a large-sample study from different universities in the country needs to be conducted.

## Conclusion

The prevalence of depression in university students was relatively high throughout our study, reaching almost one-fourth (21.8%). This indicates that a need for knowledge concerning depression still exists and should be addressed by depression-related health education programs.
